# Development of a Efficient and Sensitive Dispersive Liquid–Liquid Microextraction Technique for Extraction and Preconcentration of 10 β_2_-Agonists in Animal Urine

**DOI:** 10.1371/journal.pone.0137194

**Published:** 2015-09-08

**Authors:** Yang Li, Wei Zhang, Rui-Guo Wang, Pei-Long Wang, Xiao-Ou Su

**Affiliations:** Institute of Quality Standard & Testing Technology for Agro-Products, Chinese Academy of Agricultural Sciences, Beijing, China; CSIR- Indian Institute of Toxicology Research, INDIA

## Abstract

Dispersive liquid–liquid microextraction (DLLME) coupled with ultra-performance liquid chromatography with tandem mass spectrometry (UPLC-MS/MS) was developed for the extraction and determination of 10 β_2_-agonists in animal urine. Some experimental parameters, such as the type and volume of the extraction solvent, the concentration of the dispersant, the salt concentration, the pH value of the sample solution, the extraction time and the speed of centrifugation, were investigated and optimized. Under the optimized conditions, a good enrichment factors (4.8 to 32.3) were obtained for the extraction. The enrichment factor show that the concentration rate of DLLME is significantly higher than other pretreatment methods, and the detection sensitivity has been greatly improved. The calibration curves were linear, the correlation coefficient ranged from 0.9928 to 0.9999 for the concentration range of 0.05 to 50 ngmL^-1^ and 0.1 to 50 ngmL^-1^, and the relative standard deviations (RSDs, n = 15, intra and inter-day precision) at a concentration of 5 ngmL^-1^ were in the range of 1.8 to 14.6%. The limits of detection (LODs) for the 10 β_2_-agonists, based on a signal-to-noise ratio (S/N) of 3, were in the range of 0.01 to 0.03 ngmL^-1^. The proposed method was used to identify β_2_-agonists in three types of animal urine (swine, cattle, sheep), and the relative recoveries from each matrix were in the range of 89.2 to 106.8%, 90.0 to 109.8% and 89.2 to 107.2%, respectively.

## Introduction

β_2_-agonists (also called β_2_-receptor adrenergic agonists) are synthetic phenethanolamine compounds, which have been used in very low quantities for many years as bronchodilators for the treatment of asthma in humans and as tocolytic agents in veterinary medicine [1, 2]. They are similar in structure to the naturally occurring catecholamines dopamine, norepinephrine, and epinephrine, which are used to increase the proportion of lean meat by improving the rate of feed conversion and decreasing adipose tissue deposition in livestock. Nonetheless, there are well-documented adverse effects of β_2_-agonists on human health, such as food poisoning associated with the presence of residues in liver, cardiovascular and central nervous diseases [3, 4]. Therefore, uncontrolled use of β_2_-agonists may be dangerous for meat consumers; indeed, the use of these chemicals as growth promoters in animal diets has been banned in the United States of America by the FDA (Directives 96/22/EC and 96/23/EC), in the European Union by the Commission (EC 37/2010), and in China (The Announcement Ministry of Agriculture, No. 176, PR China, 2002). Meanwhile, because of their stimulatory activity on respiration and the central nervous system, β_2_-agonists are sometimes misused as performance enhancement drugs in horse racing and human sports [5]. The decisional threshold stated by the present International Olympic Committee Medical Commission guidelines is indeed fixed at 2 μgL^-1^ [6]. Besides, International Olympic Committee is very concerned about whether they contain β_2_-agonists residue in the animal products for athletes. It is for this reason that when sports player consume foods with β_2_-agonists residue will result in urine testing positive, and the result of athletes will be can celled. Therefore, it is desirable to develop highly sensitive analytical methods for quantitation and confirmation of trace β_2_-agonists in animal urine.

Many analytical techniques have been used for the determination of β_2_-agonists at trace levels. These methods involve an initial sample pretreatment step to isolate target analytes using liquid–liquid extraction (LLE) [7], solid phase extraction (SPE) [8, 9], QuEChERS [10], matrix solid-phase dispersion (MSPD) [11] and immunofiltration [12] followed by determination of these thermally labile compounds by liquid chromatography (LC) [13], capillary electrophoresis (CE) [14], gas chromatography (GC) [15], liquid chromatography/mass spectrometry (LC–MS) [16], gas chromatography/mass spectrometry (GC–MS) [17], liquid chromatography/tandem mass spectrometry (LC–MS/MS) [18, 19], gas chromatography/tandem mass spectrometry (GC–MS/MS) [20], ELISA [21, 22] and sensor technology [23]. However, conventional LLE uses large amounts of sample volumes and toxic organic solvents, which are often hazardous, and time-consuming to perform. Other popular sample preparation approachs, although use much less solvent than LLE, have some disadvantages as well. For example, SPE can be automated but this entails complexity and additional cost. SPME is expensive, its fiber is fragile and has limited lifetime, and sample carry-over could be a problem. QuEChERS and MSPD need special material which is expensive. Immunofiltration is affected by the matrix effect. Recently, dispersive liquid-liquid microextraction (DLLME), which was introduced by Assadi and co-workers in 2006 [24], has attracted increasing attention due its advantages of high enrichment factors, high recovery, low cost, rapid and easy operation [25]. This is a modified form of the LLE that only a microliter volume of solvent is needed to extract analytes from the aqueous samples. The key technical points is that contact area between extraction solvent and aqueous solution greatly increases. To date, numerous DLLME methods have been applied for trace level analysis in various matrices. Concerning aqueous sample analysis, DLLME has been used mainly for the extraction of organic pollutants [26–28], pesticides [29–32], insecticides [33], heavy metals [34] and illegal drugs [35]. Concerning food sample analysis, DLLME has been used mainly for the extraction of pesticides [36–38], herbicides [39], biotoxins [40, 41] and antibiotics [42, 43]. In addition, DLLME has also been used for the extraction of drugs [44, 45] and biomarkers [46] from biological samples such as blood and urine. Dispersive liquid–liquid microextraction based on solidification of floating organic drop (DLLME–SFO) [47] method has been used in determining 4 β_2_-agonists in bovine urine. In this method, β_2_-agonists were extracted by low density organic reagents, and the floating organic drop need for refrigeration to be separated from sample solution. This study presents a DLLME method does select higher density organic reagents as extraction solvent, and refrigeration is not needed. To the best of our knowledge, the application of DLLME for β_2_-agonist analysis in urine has not been described yet.

The aim of the present study was to develop and optimize DLLME conditions for the clean-up and determination of 10 β_2_-agonists in animal urine using UPLC-MS/MS. The effects of various experimental parameters on the extraction of β_2_-agonists from urine samples such as species and volume of solvent, time of extraction, salt concentration, sample solution pH, and speed of centrifugation were investigated [48]. A comparative study of present method with other reported methods was also carried out using enrichment factors bases on literature survey.

## Materials and Methods

### Reagents and standards

Ractopamine (pKa = 9.40), clenbuterol (pKa = 9.63), and bambuterol (pKa = 9.52) were purchased from Dr. Ehrenstorfer (Augsburg, Germany). Clenproperol (pKa = 9.53), tulobuterol (pKa = 9.55), and phenylethanolamine A (pKa = 9.74) were purchased from Witega (Berlin-Adlershof, Germany). Mabuterol (pKa = 9.63), cimbuterol (pKa = 9.40), brombuterol (pKa = 9.66), clorprenaline (pKa = 9.45), ractopamine d-3, and clenbuterol d-9 were purchased from Sigma–Aldrich (Berlin-Adlershof, Germany). Dichloromethane (CH2Cl_2_), chloroform (CHCl_3_), carbon tetrachloride (CCl_4_), monochlorobenzene (C_6_H_5_Cl) and ethylene dichloride (C_2_H_4_Cl_2_) (spectroscopy grade) were purchased from Sinopharm (Beijing, China). Sodium chloride and sodium hydroxide were purchased from Merck (Darmstadt, Germany). Acetone, methanol and acetonitrile of LC-grade were purchased from Merck. Ultra-pure water was obtained from a Milli-Q ultra-pure system (Millipore, Bedford, MA, USA). All the pKa were calculated by Marvin Beans software.

Stock solutions of individual β_2_-agonists containing 100 mgL^-1^ of the target compounds were prepared in methanol and stored at −20°C. Mixed standard solutions of 1 mgL^-1^ of each β_2_-agonist were prepared in methanol.

Blank urine samples were collected by veterinarians from healthy swine, cattle and sheep which were fed in our own experimental animal center with obtained permission from the animals owners. After collected the blank urine was stored in polytetrafluoroethylene (PTFE) flasks at −20°C prior to analysis.

### Instrumentation

Chromatographic separation was carried out using an ACQUITY UPLC system (Waters, Milford, MA, USA) with an ACQUITY BEH C_18_ column (100 mm×2.1 mm, 1.7 μm particle size). The column oven temperature was set at 40°C, the flow rate was at 0.3 mLmin^-1^, and a sample volume of 5.0 μL was injected by an auto sampler. The mobile phase consisted of water containing 0.1% formic acid (A) and acetonitrile (B). The initial composition was 95% A and 5% B. A gradient elution was performed in which the initial composition was 95% A, and the amount of A was decreased linearly to 50% from 0.8 to 3.0 min, then decreased to 10% over another 4.0 min, then held constant for 2.0 min and finally returned to the initial composition over 1.0 min.

Mass spectrometry was performed on a XEVO TQ mass spectrometer (Waters, Milford MA, USA) using the positive electrospray ionization mode (ESI+). The capillary voltage was set at 2.00 kV. Nitrogen was used as the nebulizing gas, desolvation gas and cone gas. The flow of the desolvation gas and cone gas was set to 800 and 50 Lh^-1^, respectively. The source and desolvation temperatures were held at 150°C and 350°C, respectively. During tandem mass spectrometric analysis, ultra-high pure argon was used as the collision gas at a flow rate of 0.13 mLmin^-1^. The retention time, declustering potential, collision energy, parent ion, and product ions for each analyte are listed in Table 1.

**Table 1:** Name of β_2_-agonists, retention time, parent ions, product ions, cone voltage, collision energy, and internal standard.

No.	Compound	Retention time	Parent ion	Product ion	Declustering Potential	Collision energy	Internal standard
		Min	m/z	m/z	V	eV	
1	Clenbuterol	4.14	277.00	203.00*/259.00	22	20/10	11
2	Cimbuterol	2.86	234.00	160.00*/216.00	21	15/9	12
3	Mabuterol	4.65	310.97	236.96*/216.96	12	16/24	11
4	Brombuterol	4.44	367.00	293.00*/349.00	24	18/12	11
5	Bambuterol	4.49	368.03	294.04*/71.89	28	18/30	11
6	Ractopamine	3.67	302.20	163.90*/284.20	25	16/12	12
7	Clorprenaline	3.79	213.97	153.94*/196.10	24	16/25	11
8	Clenproperol	3.77	263.10	245.00*/203.00	18	11/18	11
9	Tulobuterol	4.13	228.00	154.00*/172.00	30	20/20	11
10	Phenylethanolamine A	5.43	345.16	150.00*/327.00	20	22/28	11
11	Clenbuterol-d9	4.12	286.00	204.00*	22	17	-
12	Ractopamine-d3	3.66	305.30	167.11*	24	16	-

Quantification ion itemed in *.

Analytical instrument control, data acquisition, and treatment were performed using the software Masslynx version 4.1 2005 (Micro mass, Waters, Milford, MA, USA).

Optimization of the MS/MS conditions, including the choice of the ionization mode, identification of the parent and product ions, and selection of the cone and collision voltages, was performed using the individual standard solutions to provide the most favorable target analysis.

### Extraction procedure

For DLLME, the following procedure was performed: a 5.0 mL aliquot of urine was spiked with the target compounds and was placed into a 10 mL centrifuge tube; before extraction, the pH of the urine solution was adjusted to 10.0 with 2M sodium hydroxide solution; subsequently, 1.0 mL of acetone containing chloroform (100μL) was rapidly injected into the sample solution using a 1.00 mL syringe; the mixture was vortexed for 30 s, and let stand at room temperature for 10.0 minutes to enhance the extraction of target analytes from the sample solution into the tiny droplets of extraction solvent. In this final step, repeat vortex operation is needed in order to keep forming a cloudy solution (urine/acetone/chloroform). After centrifuging for 5 min at 5000 rpm, the dispersed fine droplets of chloroform sedimented at the bottom of the test tube, and the β_2_-agonists in the urine sample were extracted into the droplets (approximately 80 μL). The settled phase was withdrawn using a 100 μL microliter syringe and was then injected into the UPLC-MS/MS instrument for quantification.

## Results and Discussion

### Optimization of DLLME

In this study, DLLME combined with UPLC-ESI-MS/MS was used for preconcentration and the determination of 10 β_2_-agonists in urine samples. To develop optimized DLLME conditions, several parameters were varied for each urine sample containing 5 μgL^-1^ of each analyte. All the experiments were carried out in triplicate.

The enrichment factor (EF) was used to evaluate the extraction efficiency under different conditions. The EF was defined as the ratio between the analyte concentration in the settled phase (Csed) and the initial concentration of the analyte (C0) in the urine sample.

EF=Csed/C0

where Csed was calculated according to the external standard method.

#### Selection of dispersive solvent and extraction solvent

The most important factor affecting the selection of the dispersive solvent is its miscibility in the organic phase (extraction solvent) and the aqueous phase (urine sample) to form a distinct cloudy solution. In this study, methanol, acetonitrile and acetone, which are miscible in both the organic and aqueous phases, were tested for this purpose. The selection of an appropriate extraction solvent is of high importance and is required to achieve a high enrichment factor and selectivity of the target analytes. In classical DLLME the extraction solvent should meet six requirements: (a) higher density than water; (b) low water solubility; (c) ability to form a cloudy solution in the presence of a disperser solvent when injected into a sample solution; (d) good extraction capability of the target compounds; (e) ability to form a stable two-phase solution; (f) good chromatographic behavior. On the basis of these considerations, five chlorinated solvents, CH_2_Cl_2_ (density: 1.33 gmL^-1^), CHCl_3_ (density: 1.49 gmL^-1^), CCl_4_ (density: 1.59 gmL^-1^), C_6_H_5_Cl (density: 1.10 gmL^-1^) and C_2_H_4_Cl_2_ (density: 1.26 gmL^-1^), were examined in this research. A series of different combinations of the extraction solvent and the dispersive solvent were studied using 1.0 mL of each disperser solvent containing 100 μL of the extraction solvent. In the course of optimization, we adjusted PH value to 10.0. Then the target compounds are in a neutral form and has a higher tendency to partition itself into the organic solvent. As shown in Table 2, when methanol was used as dispersive solvent, a white turbid sediment was generated, which is not suitable for direct determination by UPLC-MS/MS. No sediment was generated when acetonitrile was used as the dispersive solvent; however, the combinations of CCl_4_-acetonitrile did sediment. However, the enrichment factors with CCl_4_-acetonitrile was at a low level. Only when acetone was used as the dispersive solvent, did each different combinations of the extraction solvent-dispersive solvent produce a clear sediment. Therefore, acetone was selected as the disperser solvent for the subsequent experiments.

**Table 2:** Settling effect of different combinations of extraction solvent-dispersive solvent.

	CH_2_Cl_2_	CHCl_3_	CCl_4_	C_6_H_5_Cl	C_2_H_4_Cl_2_
Methanol	w.t.s.	w.t.s.	w.t.s.	w.t.s.	w.t.s.
Acetonitrile	n.s.	n.s.	c.s.	n.s.	n.s.
Acetone	c.s.	c.s.	c.s.	c.s.	c.s.

w.t.s., white turbid sediment; n.s., not sediment; c.s., clear sediment

**Fig 1:**
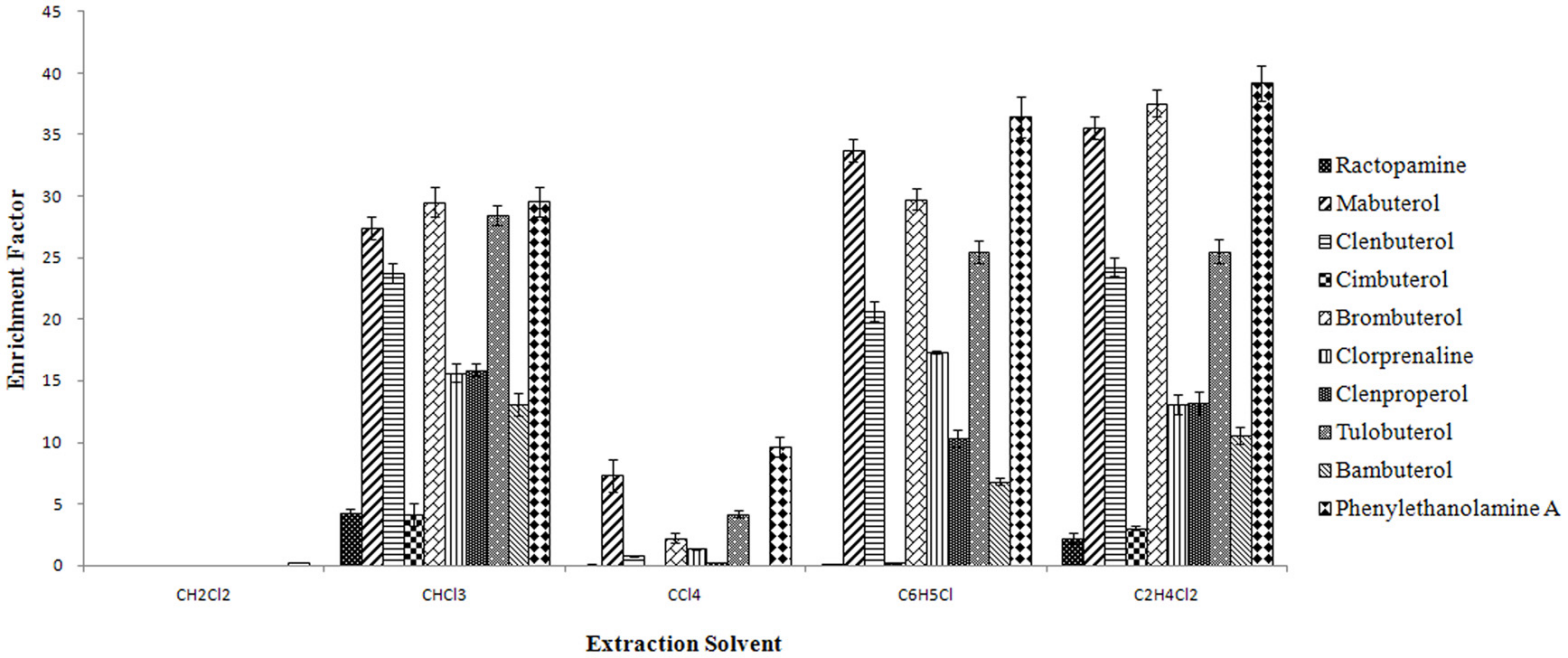
Effect of the nature of the extraction solvent on the enrichment factors of 10 β_2_-agonists.

In this study, combinations of 100 μL of CH_2_Cl_2_, CHCl_3_, CCl_4_, C_6_H_5_Cl, and C_2_H_4_Cl_2_ with 900μL of acetone as a disperser were used. The sedimented phase volume order was as follows: CHCl_3_-acetone > C_6_H_5_Cl-acetone > C_2_H_4_Cl_2_-acetone > CCl_4_-acetone > CH_2_Cl_2_-acetone. As shown in Fig 1, none of the10 β_2_-agonists with CH_2_Cl_2_ was detected, and the enrichment factors with CHCl_3_, CCl_4_, C_6_H_5_Cl, and C_2_H_4_Cl_2_ increased from 4.2 to 29.6, 0 to 9.6, 0.1 to 36.4, and 2.2 to 39.2, respectively. The results showed that the enrichment factors with CHCl_3_, C_6_H_5_Cl and C_2_H_4_Cl_2_ were higher than those of CH_2_Cl_2_ and CCl_4_. C_6_H_5_Cl was not able to preconcentrate ractopamine and cimbuterol to a desirable level. C_2_H_4_Cl_2_ was found to preconcentrate 10 β_2_-agonists; however, the enrichment factors of ractopamine, cimbuterol, clorprenaline, clenproperol and bambuterol were lower than that found for CHCl_3_. Therefore, based on the enrichment factors of the 10 β_2_-agonists, CHCl_3_ was selected as the extraction solvent in the subsequent experiment.

#### Effect of extraction solvent volume

**Fig 2:**
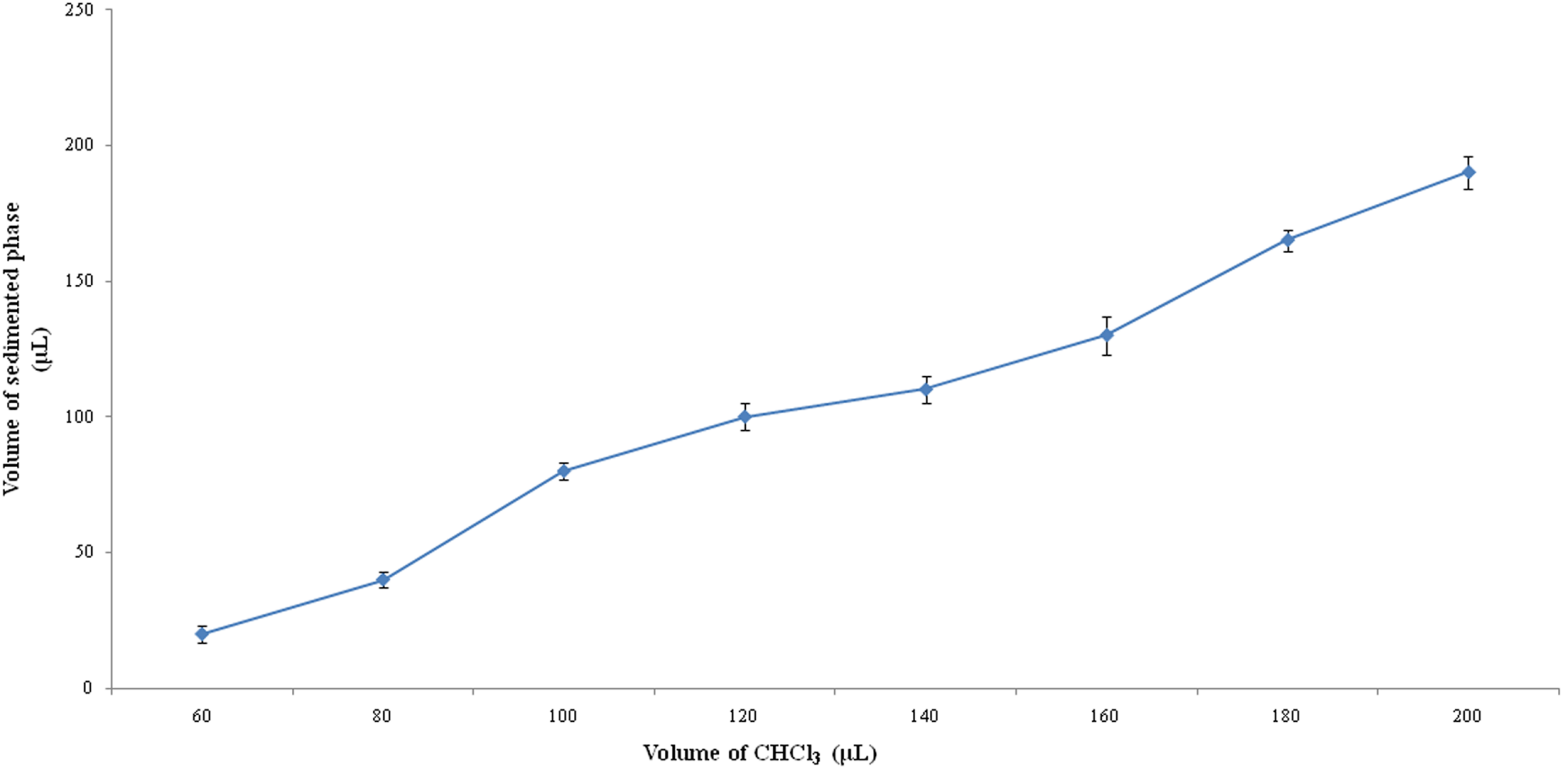
Effect of the volume of the extraction solvent (CHCl_3_) on the volume of the sedimented phase.

**Fig 3:**
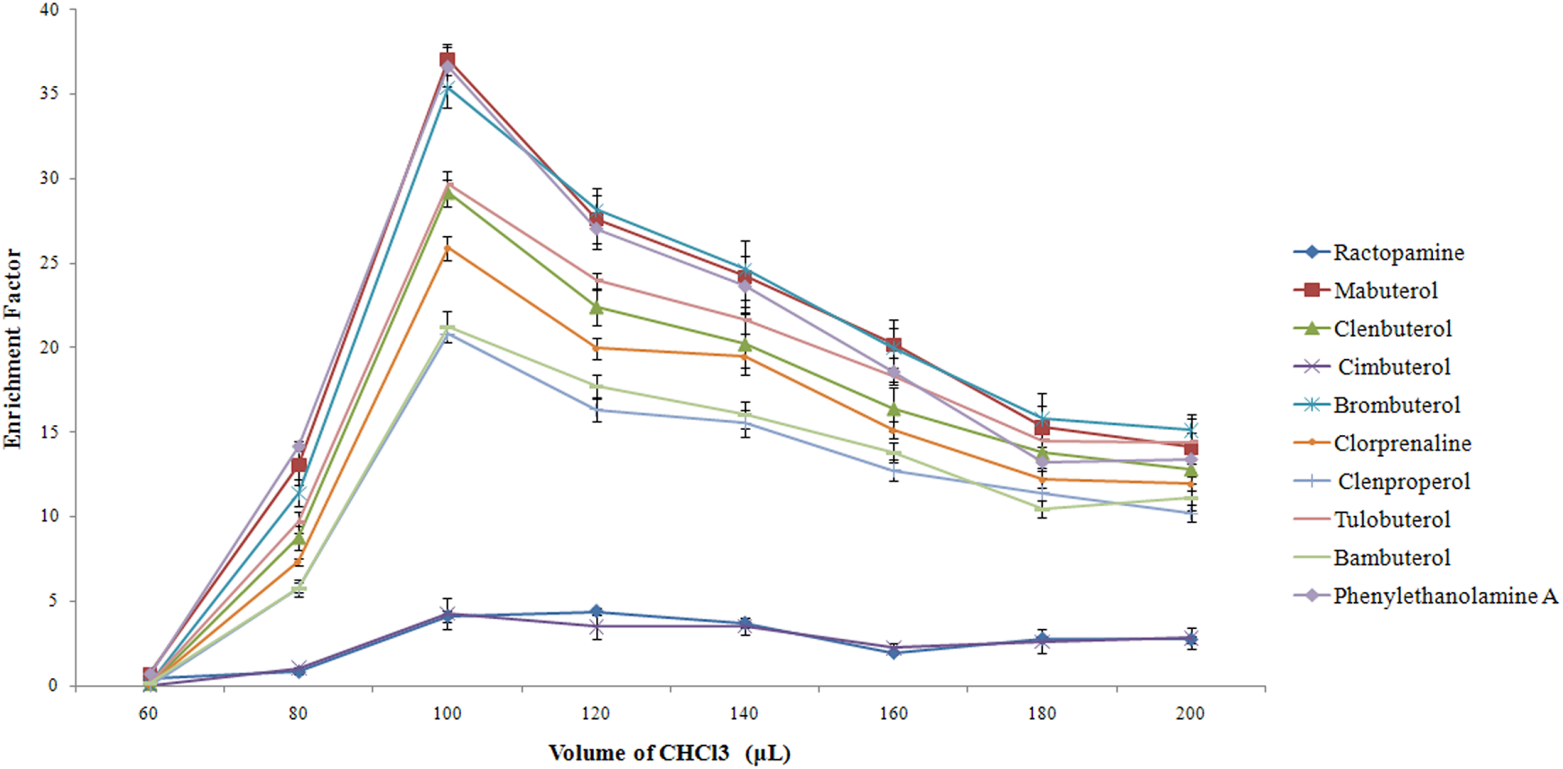
Effect of the volume of the extraction solvent (CHCl_3_) on the enrichment factors of 10 β_2_-agonists extracted by DLLME.

To evaluate the effect of the extraction solvent volume on the extraction efficiency, different volumes of CHCl_3_ (60–200 μL at 20 μL intervals) and a constant volume of acetone (1.0 mL) were tested. The results are shown in Figs 2 and 3. As the volume of CHCl_3_ increased steadily from 60 to 200 μL, the volume of the sedimented phase increased from 30 to 190 μL. The EF increased initially and then decreased after 100 μL of sedimented phase. The observed decrease was due to the fact that as the volume of the extraction solvent increases, the sedimented volume also increases, resulting in the dilution of the analytes in the extraction solvent thus leading to decreased sensitivity. On the basis of these results, 100 μL of CHCl_3_ was selected in order to obtain a high enrichment factor and a low detection limit value in subsequent experiments.

#### Effect of dispersive solvent volume

**Fig 4:**
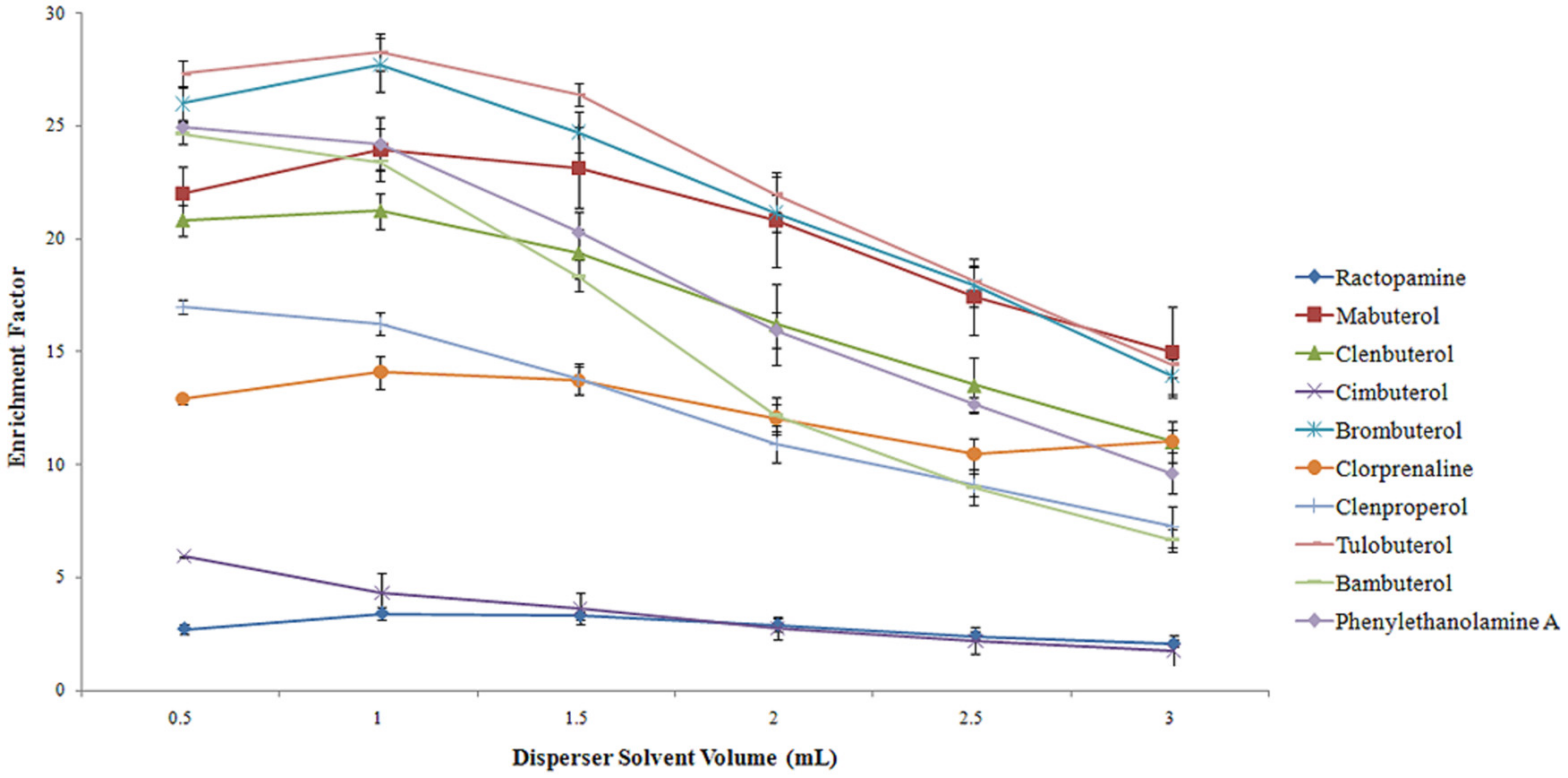
Effect of the disperser solvent volume on the enrichment factor.

The volume of the dispersve solvent directly affected the extraction efficiency. To acquire the optimal volume, experiments were performed with different acetone volumes (0.50, 1.00, 1.50, 2.00, 2.50, 3.00 mL) each containing 100 μL of CHCl_3_. As shown in Fig 4, the enrichment factors of 6 β_2_-agonists increased when the volume was less than 1.00 mL and then decreased with a further increase in the volume of acetone. The enrichment factors of other 4 β_2_-agonists decreased continually with different acetone volumes. This happened because acetone cannot disperse CHCl_3_ properly and a cloudy solution was not completely formed at a low volume. In this case, not all compounds can be extracted efficiently; however, when the volume is higher, the solubility of the β_2_-agonists in water increased. Additionally, the difference of the enrichment factors for 4 β_2_-agonists at acetone volumes of 0.50 and 1.0 mL was not significant, Therefore, the disperser solvent volume of 1.0 mL was chosen as the optimum volume for further study.

#### Effect of extraction time

In DLLME, the extraction time is defined as the interval time between injecting the mixture of the disperser solvent (acetone) and the extraction solvent (CHCl_3_) before centrifugation. In this study, the effect of the extraction time was examined within a time frame of 0–30 min while the other experimental conditions were kept constant. Repeat vortex operation is needed for different intervals of time 5, 10, 20, 30 min in order to keep forming a cloudy solution. The experimental result shows that the EF increased in 10 min, but the difference was not significant. Thus, the extraction time did not have a significant effect on the extraction efficiency. The result also showed that the transfer of the analytes from the aqueous phase to the extraction phase was very fast and the equilibrium status was quickly achieved. This is the most remarkable advantage of the DLLME technique. Therefore, because of the feasibility and maneuverability for practical use, 10 min was chosen as the extraction time.

#### Effect of salt concentration

**Fig 5:**
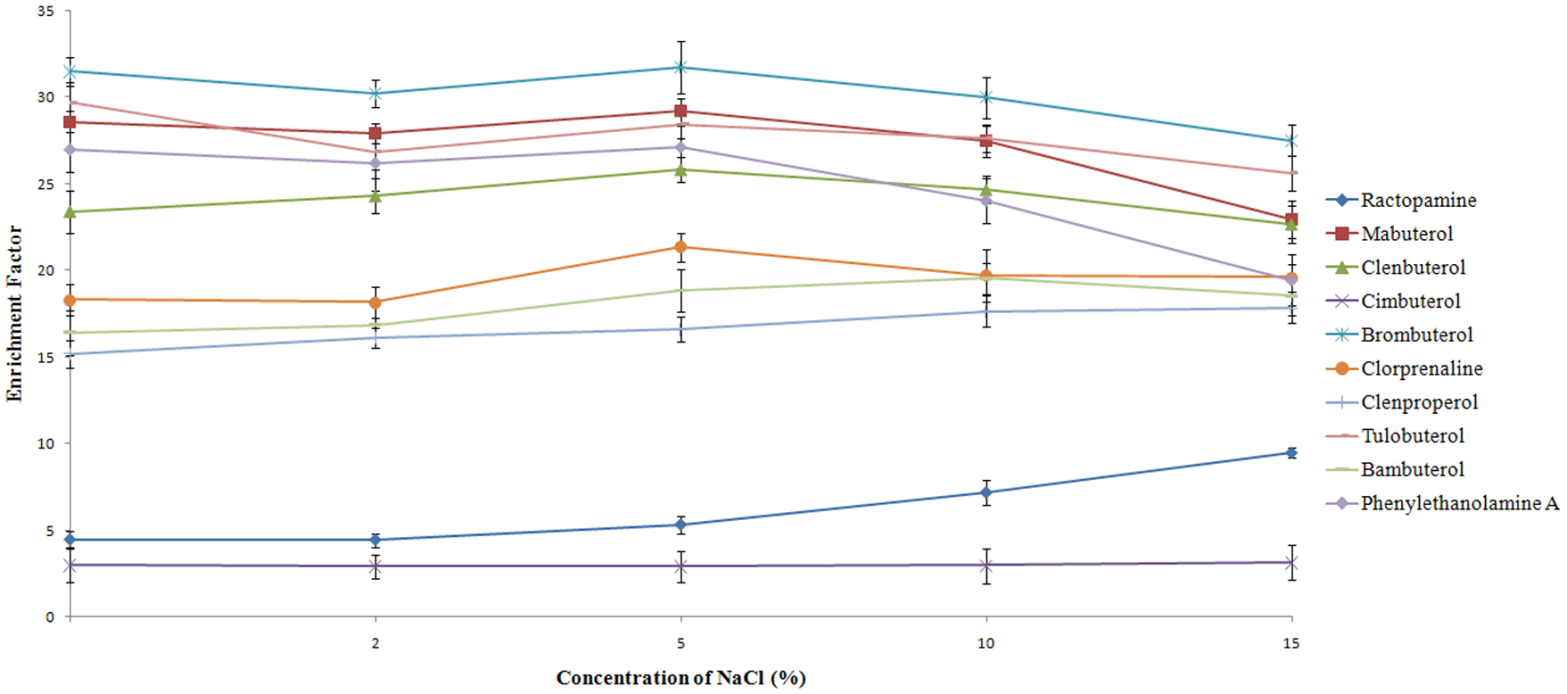
Effect of the salt concentration on the enrichment factor of 10 β_2_-agonists obtained from DLLME.

An increase in salt concentration should decrease the solubility of analytes in the aqueous sample and enhances their partitioning into the organic phase for the salting out effect. To study the effect of salt concentration on the extraction efficiency of DLLME, a series of experiments were performed by adding different amounts of NaCl (0–30%, w/v) while the other experimental conditions were held constant. The results obtained show that the extraction solvent (CHCl_3_) floated upon the aqueous solution of the urine sample after extraction and centrifugation when a concentration of 20–30% of NaCl (w/v) was added to the sample solution. The experimental result in Fig 5 shows that the extraction efficiency of the 10 β_2_-agonists depends on the salt concentration. The enrichment factor of DLLME for the extraction of some of the β_2_-agonists (e.g., mabuterol, clenbuterol, brombuterol, clorprenaline, tulobuterol, and phenylethanolamine A) increased when the concentration of NaCl increased from 0% to 5% and then decreased from 5% to 15%. Conversely, some β_2_-agonists (e.g., ractopamine, clenproperol, bambuterol) continuously increased as the concentration of NaCl increased from 2% to 15%. These observations show that the salting out effect was complicated. On the one hand, the presence of salt increases the enrichment factor by increasing the extraction efficiency. On the other hand, salting out effect increases the ionic strength of the solution, thus decreases the aqueous solubility of the extraction solvent, resulting in the increase in the volume of the sedimented phase, which in turn decreases the EF. Considering the whole process, increasing the salt concentration had no obvious impact on EF of β_2_-agonists. In addition, the concentration of salt remains constant in the urine samples itself. Therefore, salt addition was not used in subsequent experiments.

#### Effect of the sample solution pH

**Fig 6:**
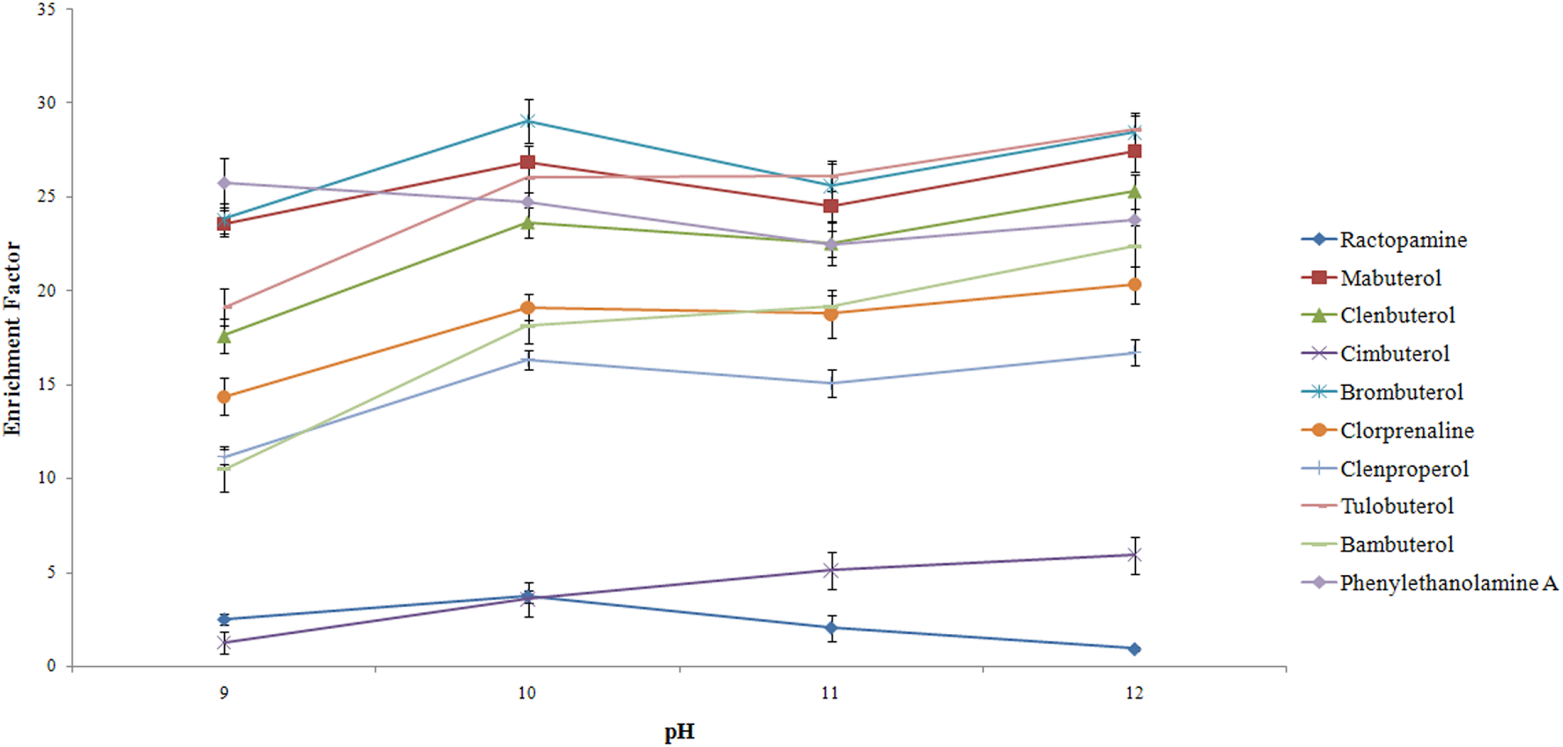
Effect of the sample pH on the enrichment factor.

The pH of the sample is an important factor that may affect the extraction efficiency of analytes in the urine samples. The β_2_-agonistsare alkaline compounds, where the pKa values are higher than 9.0 (like those shown in Reagents and standards). When the sample’s solution pH is higher than 9.0, the analyte is in a neutral form and has a higher tendency to partition itself into the organic solvent. In this study, the effect of pH on the extract ability of the β_2_-agonists with DLLME was investigated by varying the pH values from 9.0 to 12.0 with the addition of sodium hydroxide solution to the urine samples. The results shown in Fig 6 indicate that 3 extraction factors are the highest at pH 10 and the other 7 extraction factors are the highest at pH 12. However, there was no significant change at pH 10 and pH 12 to 9 target compounds. But for ractopamine which already had low EF, the extraction factor declined significantly at pH 12. In order to ensure the high extraction factors of all 10 target compounds, pH 10 was selected in the DLLME procedure.

#### Effect of centrifugation speed

The speed of centrifugation may affect the extraction solvent volume and may also help eradicate emulsification. In this research, a series of centrifugation speeds, which ranged from 1000 rpm to 5000 rpm, was studied while the other experimental conditions were kept constant. The results indicate that the enrichment factor increased slightly when the speed of centrifugation was 5000 rpm, and there was no significant change in the extraction solvent volume. Thus, a centrifugation speed of 5000 rpm was selected and used in the DLLME procedure.

#### Recoveries calculated by using internal calibration

**Fig 7:**
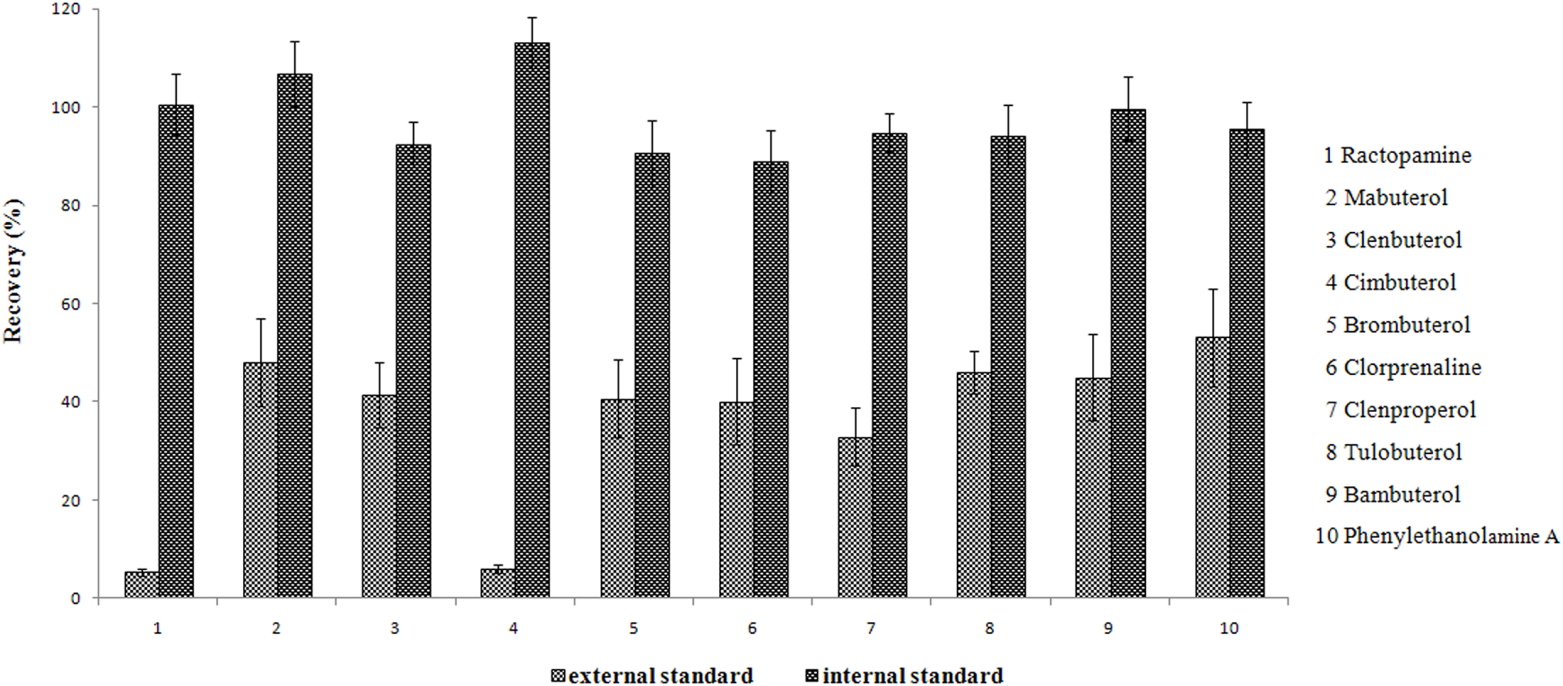
Recoveries of the 10 β_2_-agonists calculated by the internal standard method and the external standard method.

In the present study, the recoveries of the 10 β_2_-agonists were calculated by the internal standard method and the external standard method and then compared. When using the internal standard method, two internal standards, ractopamine d-3, clenbuterol d-9, were added before the DLLME procedure. The different calculated recoveries are shown in Fig 7. As observed from the results, the recoveries of the 10 β_2_-agonists calculated using external standard method were less than 50%. Additionally, the recoveries calculated using the internal standard method increased from 88.9% to 113.1%; these values were greater than those obtained with the external standards. The main reason for this result is that the target compounds cannot be extracted completely using the DLLME procedure. It is difficult for the absolute recovery calculated by the external standard method to reach 100% in theory. The loss of extraction has been overcome by using an internal standards. Therefore, the internal standard method was used to calculate the recovery of the 10 β_2_-agonists extracted by the DLLME procedure.

### Evaluation of DLLME method

#### Features of the method

**Fig 8:**
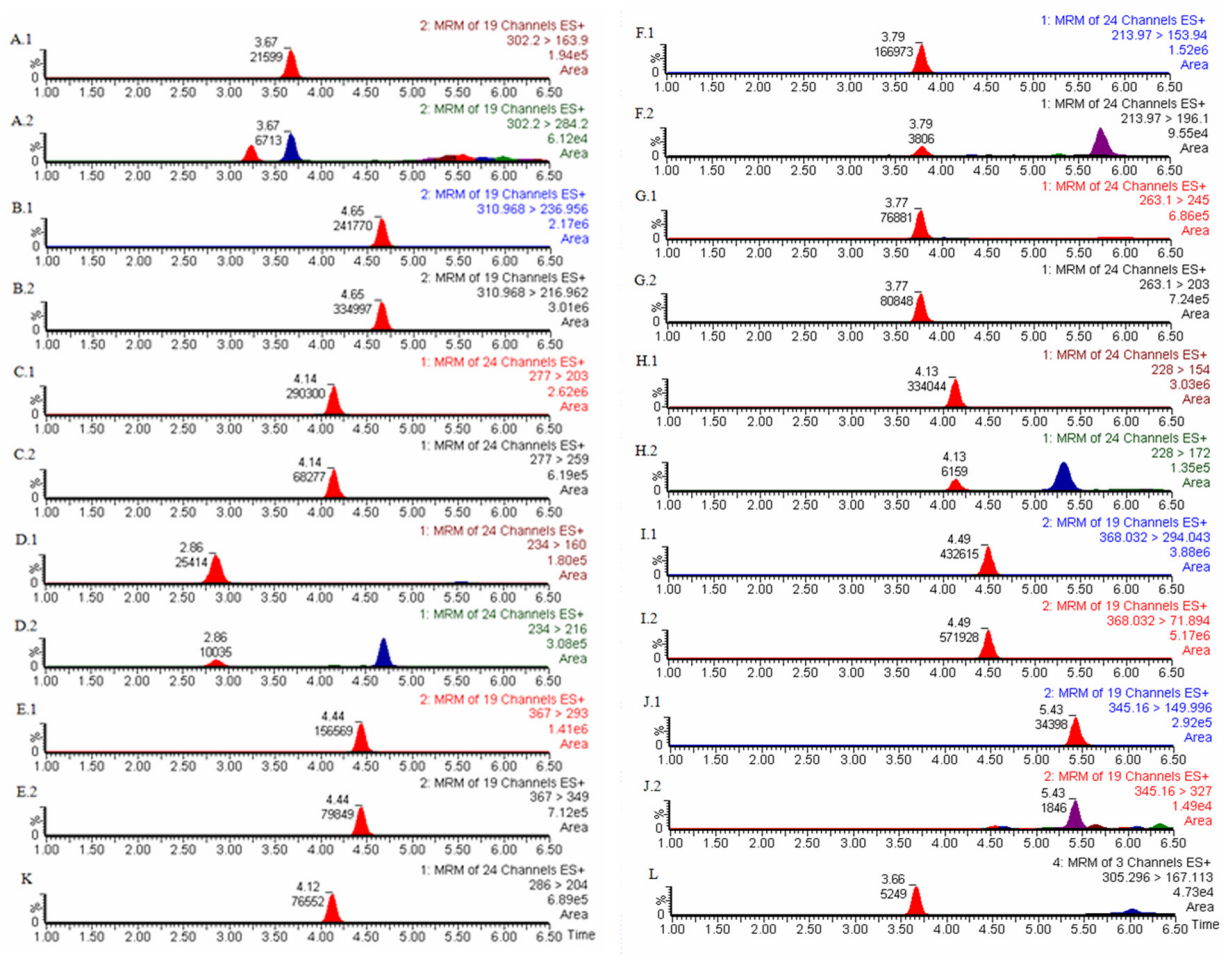
Daughter ion chromatograms of 10 β_2_-agonists.

The optimum experimental conditions were used to assess the applicability of the proposed method for quantitative determination of the target analytes by UPLC-MS/MS. The analytical characteristics of the optimized method, including linearity range, limits of detection (LODs), limits of quantification (LOQs), reproducibility (RSDs, n = 15, intra-day each six measurements, inter-day on three different days each three measurements a day) and the enrichment factor (EF), are listed in Table 3. The symmetrical peak shape chromatogram of a urine sample after spiking the 10 β_2_-agonists at a concentration of 5 ngmL^-1^ is shown in Fig 8.

**Table 3:** Linearity, reproducibility, enrichment factors LODs and LOQs of the proposed analytical procedure.

Analytes	RSDs (%) (n = 15)			EF 5 ngmL^-1^	Line arrange (ngmL^−1^)	R^2^	LODs (ngmL^−1^)	LOQs (ngmL^−1^)
	0.10 ngmL^-1^	5 ngmL^-1^	50 ngmL^-1^					
Ractopamine	10.2	14.6	6.9	5.3	0.10–50	0.998	0.03	0.1
Mabuterol	5.4	7	8	29	0.05–50	0.9993	0.01	0.05
Clenbuterol	9.2	7.5	5.4	24.5	0.05–50	0.9941	0.01	0.05
Cimbuterol	7.6	8.3	6	4.8	0.10–50	0.9972	0.03	0.1
Brombuterol	7	4.3	7.2	31.4	0.05–50	0.999	0.01	0.05
Clorprenaline	4.7	1.8	5.3	15.6	0.05–50	0.9985	0.01	0.05
Clenproperol	10.2	8.6	10	14.3	0.05–50	0.9951	0.01	0.05
Tulobuterol	6.5	4.1	7.8	32.3	0.05–50	0.9928	0.01	0.05
Bambuterol	7	6.3	6.5	15.6	0.05–50	0.9999	0.01	0.05
Phenylethan-olamine A	8.5	10.3	5.9	30.3	0.05–50	0.9994	0.01	0.05

Linearity of the calibration curve was observed in the concentration range of 0.05 to 50 ngmL^-1^ and 0.1 to 50 ngmL^-1^ with correlation coefficients ranging from 0.9928 to 0.9999. The precisions, obtained at 3 different concentration of 0.1, 5 and 50 ngmL^-1^ on one day (each six measurements) and on three different days (each three measurements a day), were in the range of 1.8% to 14.6%. The limits of detection (LODs, S/N = 3) were 0.01 to 0.03 ngmL^-1^ and the limits of quantification (LOQs, S/N = 10) were 0.05 to 0.10 ngmL^-1^. The enrichment factor of 5 ngmL^-1^ from 4.8 to 32.3.

#### Real sample analysis

According to literature [49], we have prepared blank samples and spiked samples as real sample to validate the applicability and accuracy of the proposed methods, and β_2_-agonists were extracted from three types of urine samples (swine, cattle and sheep) using the DLLME method and were analyzed by UPLC- MS/MS. The results were calculated using the internal standards clenbuterol d-9 and ractopamine d-3. Each sample was spiked with the target compounds at two different concentration levels of 0.5 and 5 μgL^-1^. The extraction procedure was repeated six times. Recoveries obtained with precision were calculated and are listed in Table 4. The results show that the analyzed urine samples were free of β_2_-agonist contamination, the recoveries were between 89.2% and 109.6%, and the RSD values were between 3.40% and 12.35% for all of the 10 β_2_-agonists in the spiked samples. The matrices of the three real types of urine samples had little effect on the proposed DLLME method for preconcentration of the β_2_-agonists from urine samples.

**Table 4:** Recovery values for three real urine samples with a spiked concentration of 0.5 and 5 ngmL^-1^ for ten β2-agonists.

	Swine Urine				Cattle Urine				Sheep Urine			
	Added (ngmL^-1^)	Found (ngmL^-1^)	Recovery (%)	RSD (%)	Added (ngmL^-1^)	Found (ngmL^-1^)	Recovery (%)	RSD (%)	Added (ngmL^-1^)	Found (ngmL^-1^)	Recovery (%)	RSD (%)
Ractopamine^a^	-	nd	-	-	-	nd	-	-	-	nd	-	-
	0.5	0.49	98	7.32	0.5	0.46	92	8.23	0.5	0.45	90	8.76
	5	5.03	100.6	6.16	5	4.78	95.6	7.8	5	4.83	96.6	10.41
Mabuterol^b^	-	nd	-	-	-	nd	-	-	-	nd	-	-
	0.5	0.5	100	8.5	0.5	0.5	100	9.77	0.5	0.51	102	10.23
	5	5.34	106.8	6.66	5	5.28	105.6	10.35	5	4.89	97.8	8.24
Clenbuterol^b^	-	nd	-	-	-	nd	-	-	-	nd	-	-
	0.5	0.45	90	4.45	0.5	0.49	98	4.37	0.5	0.48	96	4.45
	5	4.62	92.4	5.12	5	4.81	96.2	6	5	4.56	91.2	3.4
Cimbuterol^a^	-	nd	-	-	-	nd	-	-	-	nd	-	-
	0.5	0.49	98	6.98	0.5	0.5	100	5.89	0.5	0.53	106	6.54
	5	5.16	103.2	4.72	5	5.48	109.6	6.79	5	5.36	107.2	5.03
Brombuterol^b^	-	nd	-	-	-	nd	-	-	-	nd	-	-
	0.5	0.49	98	6.05	0.5	0.45	90	10.43	0.5	0.52	104	6
	5	4.52	90.4	7.64	5	4.6	92	9.16	5	5.02	100.4	5.67
Clorprenaline^b^	-	nd	-	-	-	nd	-	-	-	nd	-	-
	0.5	0.47	94	8.89	0.5	0.49	98	7.54	0.5	0.46	92	9.23
	5	4.46	89.2	6.4	5	4.74	94.8	7.66	5	4.47	89.4	12.35
Clenproperol^b^	-	nd	-	-	-	nd	-	-	-	nd	-	-
	0.5	0.46	92	6.67	0.5	0.47	94	6.75	0.5	0.48	96	4.34
	5	4.74	94.8	4.75	5	4.77	95.4	6.2	5	4.89	97.8	6.43
Tulobuterol^b^	-	nd	-	-	-	nd	-	-	-	nd	-	-
	0.5	0.45	90	5.69	0.5	0.49	98	8.02	0.5	0.48	96	5.21
	5	4.7	94	7.58	5	5.02	100.4	7.79	5	4.71	94.2	6.13
Bambuterol^b^	-	nd	-	-	-	nd	-	-	-	nd	-	-
	0.5	0.46	92	4.38	0.5	0.49	98	8.35	0.5	0.45	90	6.78
	5	4.99	99.8	6.13	5	5.27	105.4	12.19	5	4.88	97.6	5.47
Phenylethanola mine A^b^	-	nd	-	-	-	nd	-	-	-	nd	-	-
	0.5	0.47	94	5.89	0.5	0.49	98	7	0.5	0.45	90	7.96
	5	4.78	95.6	5.62	5	5.23	104.6	10.32	5	4.47	89.2	8.98

^a^. calculated using the internal standard of Ractopamine d-3.

^b^. calculated using the internal standard of Clenbuterol d-9.

#### Comparison of DLLME with other methods

The currently proposed method was compared to other methods based on the extraction and determination of the β_2_-agonists from urine samples. The enrichment factor (EF), relative standard deviation (RSD), linear range (LR), limits of detection (LODs) and extraction time for different analytes and methods are shown in Table 5. As observed in Table 5, the shortest extraction time was observed for the DLLME method, which was less than 15 min, whereas the extraction time for the LLE, SPE, QuEChERS and MSPD methods ranged from 30 to 60 min. The RSDs for the DLLME method was between the RSD values for the other methods. A good linear range (0.05–50 ngmL^-1^) and sensitive LODs (0.01–0.03 ngmL^-1^) were observed for this method. The volume of the sample solution required for DLLME is approximately 5 mL, which is similar to that of the LLE, SPE, QuEChERS and MSPD methods. Additionally, the DLLME method required lower solvent volumes and did not require a special approach or instrument in the pretreatment step.

**Table 5:** Comparison of DLLME to other methods for the determination of β_2_-agonists.

Method	Analyte	EF	RSD (%)	LR (ngmL^−1^)	LOD (ngmL^−1^)	Extraction time (min)	Reference
LE-LC-MS-MS	clenbuterol	66	≤10.6	0.05–3	0.05	30	[7]
PE-LC-MS-MS	3 β_2_-agonists	15	≤10.5	0.5–5	0.5	60	[8]
QuEChERS-LC-MS/MS	14 β_2_-agonists	5	<16.6	10–250	1.8–23.1	30	[10]
MSPD-LC-IT-MS	clenbuterol	25	-	0.5–100	0.1	30	[11]
DLMLE-LC-MS-MS	10 β_2_-agonists	4.8–32.3	≤12.3	0.05–50	0.01–0.03	15	Represented method

## Conclusions

In the present study, DLLME extraction pretreatment coupled with the UPLC- MS/MS method has been successfully applied to the simultaneous determination of 10 β_2_-agonists in urine samples. This technique provides low limits of detection, good repeatability and good recovery within a short testing time. Comparing this with other methods, DLLME is simple, rapid, sensitive, inexpensive and environmentally friendly, which are among the strongest advantages. Nevertheless, this procedure also has some limitations, such as the requirement for chlorine-bearing reagents. Therefore, a larger volume of washing fluid should be used to avoid corrosion of the metal injection needle of the auto-sampler.

Furthermore, centrifugation is required before extraction by DLLME when it contains a lot of solid impurities in urine. The excellent performance profile of the DLLME method for the analysis of real urine samples demonstrates the possibility of its usage in routine analysis.

## Supporting Information

S1 Fig Schematic diagram of DLLME method. (TIF)

S2 Fig Effect of the extraction time on the enrichment factor. (TIF)

S3 Fig Effect of the centrifugation speed on the enrichment factor. (TIFF)

S1 Table Mean determined peak area of different spiked concentration of 10 β_2_-agonists. (DOCX)
