# Supplementary figures and images for: Development of a Efficient and Sensitive Dispersive Liquid–Liquid Microextraction Technique for Extraction and Preconcentration of 10 β2-Agonists in Animal Urine
Source: PLoS One. 2015 Sep 8;10(9):e0137194. doi: 10.1371/journal.pone.0137194 (PMC4562641; doi:10.1371/journal.pone.0137194)

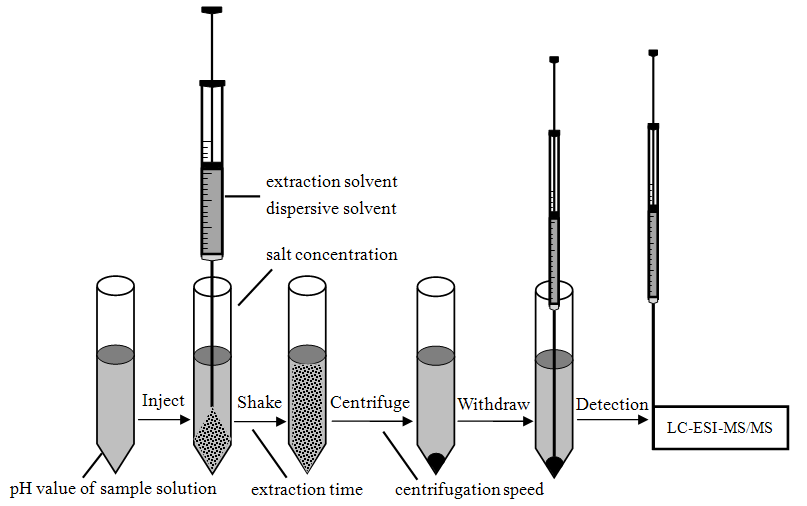

Supplement: S1 Fig — (TIF) [file pone.0137194.s001.tif]

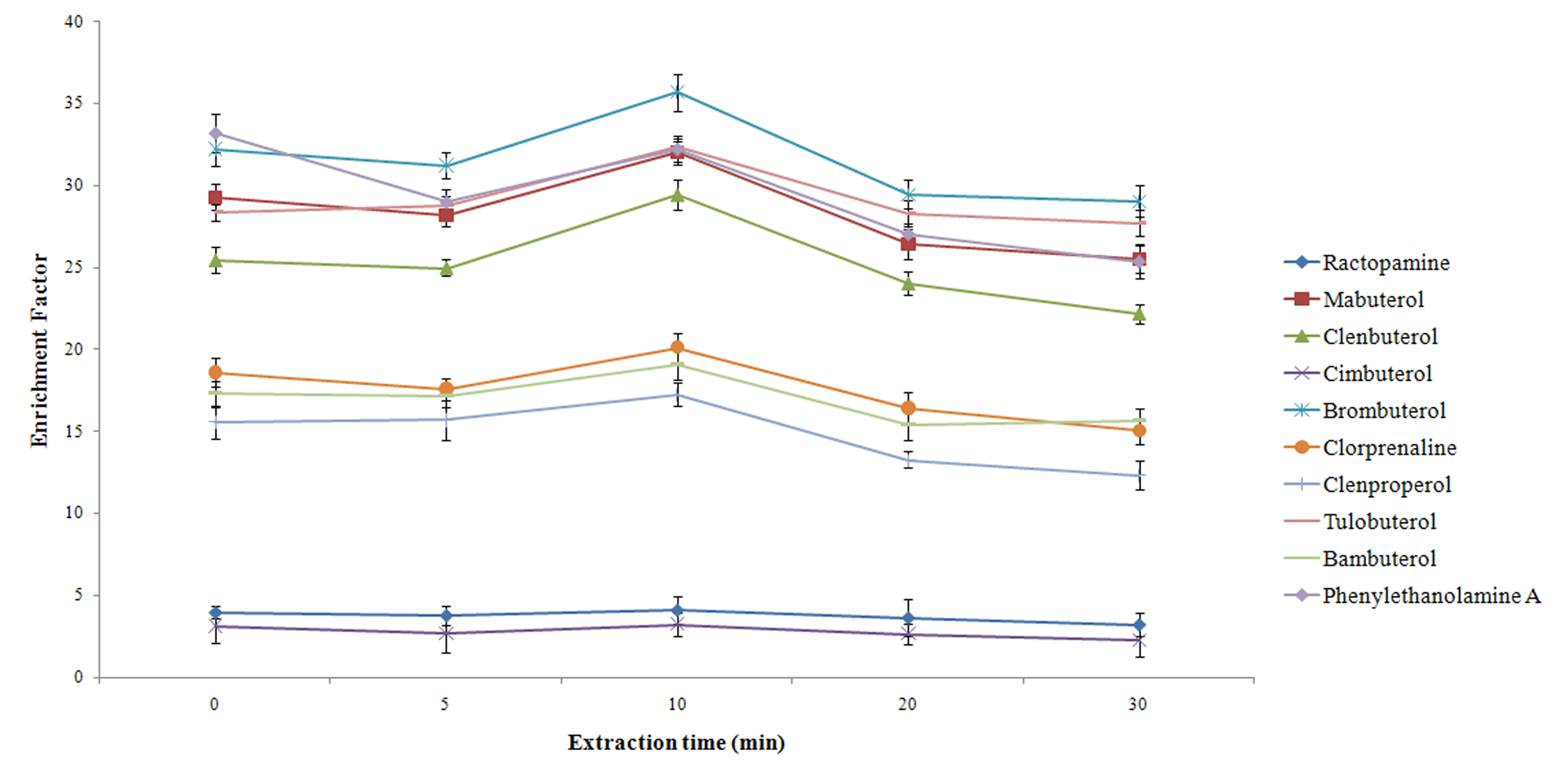

Supplement: S2 Fig — (TIF) [file pone.0137194.s002.TIF]

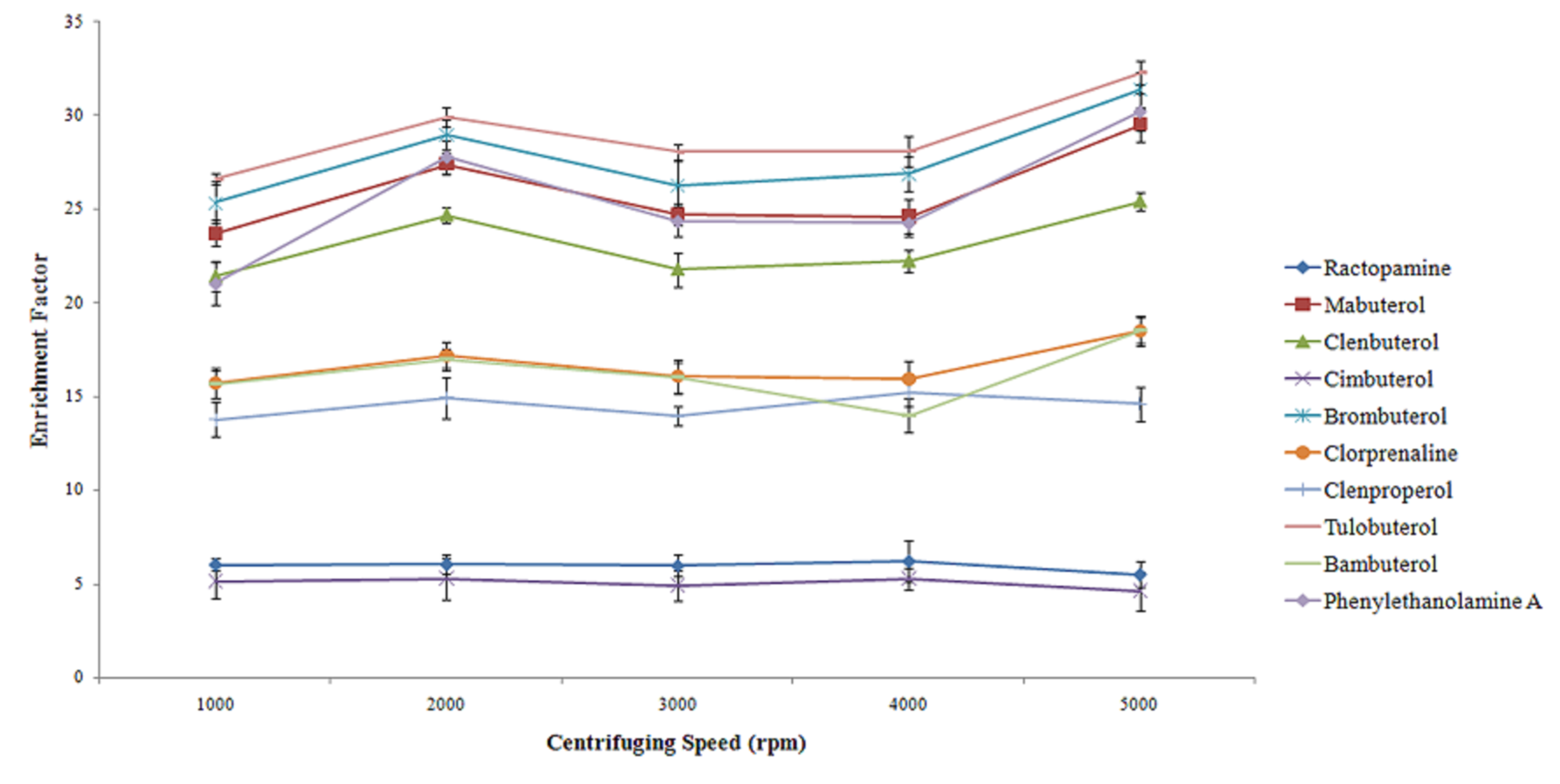

Supplement: S3 Fig — (TIFF) [file pone.0137194.s003.tiff]
