# Supplementary material for: Development of a Efficient and Sensitive Dispersive Liquid–Liquid Microextraction Technique for Extraction and Preconcentration of 10 β2-Agonists in Animal Urine
Source: PLoS One. 2015 Sep 8;10(9):e0137194. doi: 10.1371/journal.pone.0137194 (PMC4562641; doi:10.1371/journal.pone.0137194)
Supplement: S1 Table — (DOCX) [file pone.0137194.s004.docx]

| Analytes | Spiked concentration (ngmL-1) | Mean determined  peak area | Analytes | Spiked concentration (ngmL-1) | Mean determined Peak area |
| --- | --- | --- | --- | --- | --- |
| Ractopamine | 0.1 | 6472.051 | Cimbuterol | 0.1 | 2984.953 |
|  | 0.5 | 30285.82 |  | 0.5 | 16027.33 |
|  | 1 | 50202.58 |  | 1 | 31525.3 |
|  | 5 | 180918.4 |  | 5 | 143519.6 |
|  | 50 | 1240052 |  | 50 | 1081080 |
| Mabuterol | 0.05 | 1467.125 | Tulobuterol | 0.05 | 2047.753 |
|  | 0.1 | 3468.113 |  | 0.1 | 8197.807 |
|  | 0.5 | 17908.92 |  | 0.5 | 29575.46 |
|  | 1 | 34961.16 |  | 1 | 60837.24 |
|  | 5 | 171587.5 |  | 5 | 276196.7 |
|  | 50 | 1337404 |  | 50 | 1464382 |
| Clenbuterol | 0.05 | 3133.58 | Brombuterol | 0.05 | 100.864 |
|  | 0.1 | 6472.051 |  | 0.1 | 347.348 |
|  | 0.5 | 24285.82 |  | 0.5 | 1481.965 |
|  | 1 | 50202.58 |  | 1 | 2543.517 |
|  | 5 | 223918.4 |  | 5 | 17025.68 |
|  | 50 | 1240052 |  | 50 | 114932.1 |
| Clorprenaline | 0.05 | 1070.188 | Bambuterol | 0.05 | 2212.261 |
|  | 0.1 | 2914.695 |  | 0.1 | 6554.851 |
|  | 0.5 | 12670.17 |  | 0.5 | 29706.25 |
|  | 1 | 25553.42 |  | 1 | 56352.08 |
|  | 5 | 122089.4 |  | 5 | 298408.6 |
|  | 50 | 868261.5 |  | 50 | 2787634 |
| Clenproperol | 0.05 | 996.932 | Phenylethano-lamine A | 0.05 | 158.358 |
|  | 0.1 | 1027.998 |  | 0.1 | 195.246 |
|  | 0.5 | 7234.64 |  | 0.5 | 2643.111 |
|  | 1 | 13146.36 |  | 1 | 4634.771 |
|  | 5 | 60830.8 |  | 5 | 28275.7 |
|  | 50 | 350940.3 |  | 50 | 228230.9 |
